# Hounsfield Unit characterization and dose calculation on a C‐arm linac with novel on‐board cone‐beam computed tomography feature and advanced reconstruction algorithms

**DOI:** 10.1002/acm2.70145

**Published:** 2025-07-25

**Authors:** Kenneth W. Gregg, Theodore Arsenault, Atefeh Rezaei, Rojano Kashani, Lauren Henke, Alex T Price

**Affiliations:** ^1^ Department of Radiation Oncology University Hospitals Seidman Cancer Center Cleveland Ohio USA; ^2^ Department of Biomedical Engineering Case Western Reserve University Cleveland Ohio USA; ^3^ School of Medicine Case Western Reserve University Cleveland Ohio USA

**Keywords:** dose calculation, fast cone‐beam CT, Hounsfield Unit characterization, inhomogeneity correction

## Abstract

**Purpose:**

Current cone‐beam computed tomography (CBCT) on‐board c‐arm linear accelerators (linacs) lack CT number precision sufficient for dose calculation due to increased scatter from the cone geometry. This investigation evaluated CT number and dose calculation accuracy in‐phantom on a novel on‐board CBCT unit with potential for improved dose calculation accuracy.

**Methods:**

Eight head and eight body configurations of an electron density phantom using 16 materials were acquired with a clinical CT‐simulator(CT‐sim) and novel on‐board CBCT imager. CBCT projection data was reconstructed using conventional Feldkamp–Davis–Kress(FDK) and patient scatter‐corrected iterative Acuros CTS with metal artifact reduction (Acuros‐CTS‐iCBCT‐MAR) to create a robust CT number to physical density curve. CT‐sim and CBCT images of anthropomorphic head and thorax phantoms were acquired, and three treatment plans were generated per phantom. All CBCT images were registered to the CT‐sim of the phantoms, and the dose was recalculated on the CBCT images. 3D gamma analysis was performed (10% dose threshold, local, 1% dose difference, 1 or 2 mm distance to agreement), and dose–volume histogram (DVH) metrics were reported for target coverage and organ sparing.

**Results:**

CT numbers for materials ≤1.08 g/cc showed high agreement between CT‐sim and CBCT acquisitions. CT number precision improved with Acuros‐CTS‐iCBCT‐MAR compared to FDK in all materials. High agreement between CT‐sim and CBCT reconstructed with Acuros‐CTS‐iCBCT‐MAR was observed in 3D gamma analysis showing 93.8% voxels passing in the worst case for a spine stereotactic body radiotherapy (SBRT) plan at 1%/1 mm. Maximum deviation in target coverage was −3.3% PTV‐D98% in the lung SBRT plan among the novel reconstructed CBCT images. Plan comparison using FDK reconstructions yielded similar or worse agreement in 3D gamma analysis, with 69.7% voxels passing in the worst case for a spine SBRT treatment plan. Maximum target coverage deviation was −11.7% PTV‐D98% among the FDK‐reconstructed CBCT.

**Conclusions:**

Novel CBCT solutions on c‐arm linacs show promise for enabling direct‐to‐unit or offline adaptive dose calculation, potentially increasing versatility and efficiency of patient‐centered care.

## 1 INTRODUCTION

Two notable advancements in radiation oncology were the introduction of computed tomography simulation (CT‐sim) and on‐board cone‐beam computed tomography (CBCT).^1^, ^2^ CT‐sim enabled personalized treatment planning with increased dose calculation accuracy via inhomogeneity corrections using Hounsfield Unit (HU) to electron density ($\rho_{e}$) tables.^3^ Integration of CBCT in image‐guided radiation therapy (IGRT) enabled accurate patient alignment to treatment volumes via registration to the CT‐sim image for treatment. Modern IGRT workflows using CBCT offer increased internal precision compared to prior techniques such as aligning to surface markings.^4^ The associated decrease in localization uncertainty has enabled smaller treatment planning margins and reduced critical radiotoxicity to nearby organs at risk (OARs).^5^, ^6^, ^7^, ^8^

Conventional CT‐sim uses a fan‐beam geometry that rotates about a longitudinal axis to acquire projections while translating the patient parallel to this axis, resulting in a helical motion with respect to the patient that minimizes out‐of‐plane scatter while acquiring a large longitudinal extent of the patient. In contrast, CBCT acquires 2D projection images with a half‐fan cone‐beam geometry using one gantry rotation (360°) or less (200°) if a full‐fan cone‐beam geometry is chosen. In these geometries, the increased scatter contribution from the large volume irradiated in each projection results in greater noise and decreased precision and accuracy of HU values compared to fan‐beam CT (FBCT).^9^ The high HU uncertainty due to this increased scatter contribution compared to FBCT results in reduced accuracy and precision of dose calculations.^10^, ^11^

O‐ring gantry radiotherapy systems such as the Halcyon treatment platform with HyperSight (Varian Medical Systems, Palo Alto CA, USA) have demonstrated reduced noise and increased image quality over prior CBCT systems both in phantom and in vivo due to improved image panel design, advanced reconstruction methods, and faster gantry speeds.^12^, ^13^, ^14^ Although initially used for improved structure delineation in adaptive workflows, the highly improved imaging has brought to question if modern CBCT could allow for accurate calculation of radiation dose without the use of conventional CT‐sim. Although these imaging improvements in an O‐ring gantry system have been previously characterized, there has yet to be extensive characterization of the improvements on a C‐arm gantry system, such as the TrueBeam equipped with HyperSight (Varian Medical Systems, Palo Alto CA, USA). Therefore, HU evaluation and dose calculation accuracy assessment in heterogeneous phantoms using CBCTs from the improved imaging panel and advanced image reconstruction algorithms on a C‐arm linac are needed.

This work tests whether a C‐arm linac equipped with an improved CBCT imaging system and image reconstruction algorithms could be used for accurate dose calculation. Preliminary investigations evaluated how differing imaging protocols impact HU‐to‐physical density curves. Then, the dose calculation accuracy was assessed on anthropomorphic phantom geometries acquired using CT‐sim and CBCT.

## 2 METHODS

### 2.1 Imaging system description

In this study, the C‐arm TrueBeam v4.1 utilized a faster gantry speed than previous versions (1.5 rpm or 9°/s) and was equipped with a HyperSight imaging panel. Characteristics of this panel that differ from previous solutions include an improved imaging panel grid ratio (15:1) for increased scatter rejection, a larger maximum imaging area (43 × 43 cm^2^), and increased collection efficiency. Additionally, in contrast with the stationary imaging panel configuration in the Halcyon equipped with HyperSight, the C‐arm panel is capable of moving from full‐fan to half‐fan exposure geometries, changing the scatter conditions per imaging protocol.^15^ In preparation for this investigation, five scan protocols were calibrated following vendor‐supplied guidelines using the Quart phantom to correctly calculate HU values during scanning acquisition.^16^

Two sets of reconstruction algorithms were applied to the raw datasets in this investigation. First, the conventional Feldkamp–Davis–Kress (FDK) algorithm.^17^ Second, a vendor‐supplied iterative Acuros CBCT with adaptive metal artifact reduction (iCBCT MAR) reconstruction method.^18^, ^19^ FDK reconstructions are generally less computationally expensive compared to iterative algorithms, relying primarily on filtered back‐projection assuming a point source and point voxels to reconstruct an image. In FDK, scatter contributions from the irradiated volume generally degrade the image quality by increasing noise. FDK reconstructions are also susceptible to common artifacts, for example, beam hardening and photon starvation, which may reduce HU value accuracy and precision.

The second reconstruction method utilizes iterative CBCT with Acuros CTS reconstruction algorithms and hardware scatter correction (HWSC) obtained from Monte Carlo modeling of the imaging chain (available in TrueBeam v4.1). The HWSC includes corrections for off‐focal radiation, position‐dependent detector scatter, and bowtie filter scatter. Monte Carlo simulations also characterized the energy dependency of the panel for refinement of the x‐ray tube spectrum to improve imager efficiency. Bowtie motion estimation during gantry rotation was also adjusted for imaging compensation improvements specific to TrueBeam. If the adaptive metal artifact reduction algorithm does not detect the presence of metal, it defaults to the standard iterative CBCT with Acuros CTS and HWSC. This reconstruction method was chosen due to the presence of metal material rods in the phantom to create differing scattering conditions as described below.

### 2.2 HU characterization

The HU value corresponding to each material's physical density for the development of further detailed density curves was determined using the advanced electron density phantom (Sun Nuclear Corporation, Melbourne, FL, USA, S1) using the 30 × 40 cm diameter ovoid and 20 cm diameter cylindrical head section. The densities and associated materials used in the electron density phantom are presented in Table 1. First, for the reference baseline, images of the electron density phantom were acquired with a Big Bore CT‐simulator (Philips Healthcare, Andover, MS, USA) with a fan beam geometry. The CT‐sim acquisition parameters used in this analysis are described in Table 2. Second, for comparison, the electron density phantom was imaged with the CBCT. Due to the varying possible acquisition parameters and geometric techniques using the CBCT, multiple protocols were used to acquire images of the electron density phantom. These protocols are also described in Table 2. Additionally, due to the noted uncertainty in HU values with a CBCT system due to scattering conditions, eight total arrangements of the density material rods were imaged to capture HU values under differing scatter conditions. Four arrangements included density material rods up to the most dense bone‐density material (*ρ* ≤ 1.93 g/cc). Two arrangements excluded all high‐density materials (*ρ*≥1.08 g/cc). The last two arrangements included three metallic rods, which were aluminum, titanium, and stainless steel as well as omitting the bone‐density materials. This set of eight arrangements was acquired with both the CT‐sim and CBCT. Finally, all images were inserted into RapidCHECK (Sun Nuclear Corporation, Melbourne, FL, USA), which automatically identifies the density material rods and reports the descriptive statistics of the HU values within that material density rod for the 11 center‐most slices.

**TABLE 1:** List of materials for characterizing CT number to physical density mapping with specified physical density.

Material name	Physical density (g/cc)
Air	0.00
LN300 Lung	0.29
LN450 Lung	0.45
HE General Adipose	0.96
HE Breast 50/50	0.98
True Water	1.00
HE CT Solid Water	1.02
HE Brain	1.05
HE Liver	1.08
HE Inner Bone	1.21
CaCO_3_ – 30% Bone	1.33
CaCO_3_ – 50% Bone	1.56
HE Cortical Bone	1.93
Aluminum	2.71
Titanium	4.51
Stainless Steel	8.00

Abbreviation: CT, computed tomography.

**TABLE 2:** Summary of computed tomography (CT) and cone‐beam CT (CBCT) acquisition parameters used for treatment planning.

Scanner type	Protocol name	Voltage (kV)	Exposure (mAs)	Exposure geometry	Scan angle subtended	Slice thickness (mm)	Phantom geometry
CT simulator	3D/IMRT pelvis	120	119	Full‐fan	n/a	2	Body+Head
	3D/IMRT pelvis*	140	86	Full‐fan	n/a	2	Body+Head
	SBRT brain	120	591	Full‐fan	n/a	2	Head section
	SBRT brain*	100	505	Full‐fan	n/a	2	Head section
CBCT	Thorax	125	265.2	Half‐fan	360°	2	Body+Head
	Pelvis	125	1060.8	Half‐fan	360°	2	Body+Head
	Pelvis large	140	1679.6	Half‐fan	360°	2	Body+Head
	Head	100	149.7	Full‐fan	200°	2	Head section
	Head*	125	265.2	Full‐fan	360°	2	Head section

*Note*: * denotes customized scan parameters.

Abbreviations: CBCT, cone beam computed tomography; CT, computed tomography; IMRT, intensity‐modulated radiotherapy; SBRT, stereotactic body radiotherapy.

With the HU value descriptive statistics defined for each image and the reconstruction of the electron density phantom calculated, the HU values specific to each material were combined across all arrangements from the imaging protocol in aggregate separately on the CT‐sim and CBCT. Subsequently, the average and standard deviation were calculated across all arrangements for each protocol and reconstruction. The average CBCT HU values were compared to the average CT‐sim HU values by taking the difference in values. Combined standard deviation was used to assess the uncertainty of the density material rod with that imaging system. HU values were also grouped within similar materials for further comparisons: low‐density materials (true air, LN300 lung, LN450 lung), near‐water density materials (brain, breast 50/50, CT solid water, general adipose, liver, true water), and high‐density materials (CaCO3 – 30% bone, CaCO3 – 50% bone, inner bone, cortical bone).

### 2.3 Dose calculation

To test the impact of calculating the dose on a CBCT, two separate anthropomorphic phantoms were used to evaluate the differences in dose calculation metrics. The two anthropomorphic phantoms used were a head and neck phantom (CyberKnife Head and Neck Phantom, Accuray, Sunnyvale, CA, USA, S1) and a heterogeneity thorax phantom (E2E SBRT Phantom, Sun Nuclear Corporation, Melbourne, FL, USA, S1).

On each anthropomorphic phantom, three sets of treatment plans were created on the CT‐sim image to test dose calculation in varying scenarios. Organ‐at‐risk and GTV contours were drawn with PTV expansions incorporated to simulate clinically realistic scenarios. Intensity‐modulated radiotherapy (IMRT) plans were inverse‐planned by a board‐certified medical physicist in RayStation (RaySearch Laboratories, Stockholm, SE) using the Collapsed Cone Convolution (CCC, v. 5.7) dose calculation algorithm. For the head phantom, a head and neck intensity‐modulated radiotherapy (IMRT), a hippocampal‐avoidance IMRT, and a single metastases stereotactic radiosurgery (SRS) plan were created with 70 Gy in 35 fractions, 30 Gy in 10 fractions, and 27 Gy in 3 fractions, respectively. For the thorax phantom, a lung stereotactic body radiotherapy (SBRT), a spine SBRT, and a rib SBRT plan were created with dose schemes 50 Gy in five fractions, 35 Gy in five fractions, and 27 Gy in three fractions, respectively. For the CT‐sim plans, the mass‐density curve assigned was from the clinical system with the corresponding kVp. These plans created on the CT‐sim image were then transferred onto registered CBCT images acquired with the same imaging protocols and reconstructions from the HU evaluation.

For dose calculation, each CBCT protocol and reconstruction method were assigned a mass–density curve derived from the average value across all scans using that protocol and reconstruction method. For the head and neck phantom, only the full‐fan image geometries were evaluated (head and head custom). For the thorax phantom, only the half‐fan geometries were evaluated (thorax, pelvis, and pelvis large). For dose calculation comparisons on CT‐sim and CBCT, these images were rigidly registered, then 3D gamma analysis was performed and dose–volume histogram (DVH) metrics were evaluated. D98%, D50%, and D2% were reported for the planning target volume (PTV) or PTV evaluation structure and surrounding nearby organs‐at‐risk (OAR). The nearby OARs varied based on the treatment plan and are reported in Table 2. A successful DVH evaluation metric on the CBCT image was a 3% difference from the CT‐sim image using the Medical Physics Practice Guidelines (MPPG) 5b recommendation for VMAT evaluation.^20^ The gamma criteria of 1%/1mm or 1%/2 mm, each with 10% threshold, local normalization for the gamma evaluation were chosen as restrictive criteria allowed by TG‐219 and from contemporaneous studies.^20^, ^21^, ^22^ The more restrictive evaluation criteria were performed to isolate where the spatial deviations occur within a treatment plan.

## 3 RESULTS

### 3.1 HU characterization

**FIGURE 1:**
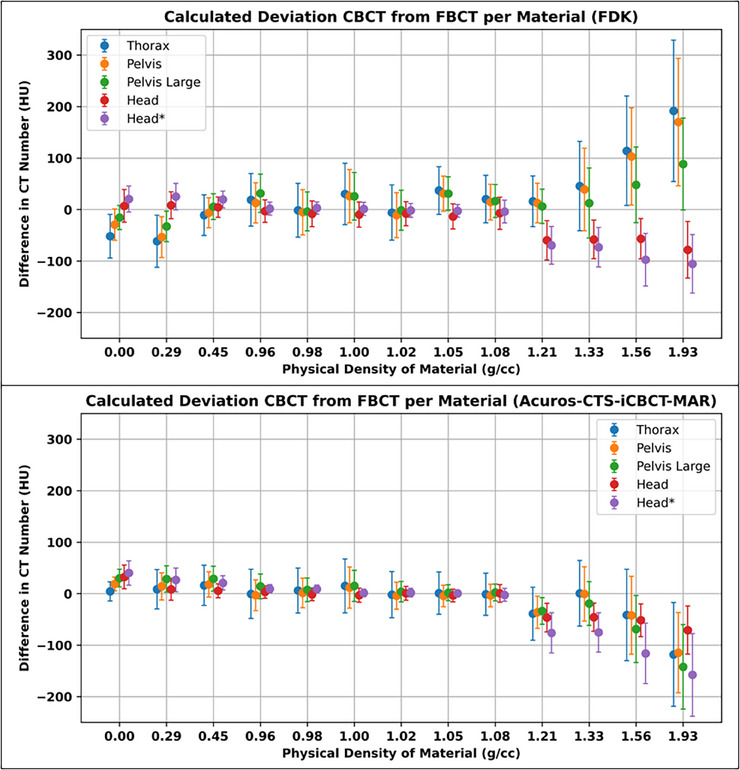
Mean difference in CT number averaged across all phantom and rod configurations per CBCT protocol substracted from FBCT average with nearest corresponding kVp. Error bars repersent combined standard deviation across all scans calculated per protocol and material. CT, computed tomography; CBCT, cone beam computed tomography; FBCT, fan beam computed tomography; kVp, kiloVolt peak.

The HU value differences across varying densities and protocol techniques are illustrated in Figure 1. Each material CT number from CBCT protocol was subtracted from the closest valued kVp technique. The FDK protocols had larger deviations from the FBCT compared to the iCBCT protocols when evaluating HU values in the near‐water density ranges. The average HU value difference for the FDK near‐water density materials was 7.2 ± 15.4HU compared to 2.6 ± 5.7HU for iCBCT. For the low‐density and high‐density materials, FDK had greater variance compared to the iCBCT protocols. In particular, for the FDK reconstructions, the two fan types deviated further and opposite about the 0% difference point. This resulted in HU values averaging closer to zero for the FDK protocols despite being further away from the CT‐sim HU value. For example, for FDK, the average HU value difference for the low‐density materials was ‐11.4 ± 27.5HU compared to 20.1 ± 10.4HU for the iCBCT protocols. Similarly, the HU value density for the high‐density materials was 12.5 ± 85.9HU for FDK whereas it was −64.7 ± 43.5HU for iCBCT. When performing absolute value averages, the average HU value difference for the low‐density materials with an FDK reconstruction was 23.5 ± 18.3HU compared to 20.1 ± 10.4HU for the iCBCT protocols. Similarly, for high‐density values, the average HU value difference was 72.3 ± 48.0HU compared to 64.8 ± 43.4HU. For near‐water densities, these differences for FDK and iCBCT were 12.9 ± 11.0HU and 4.5 ± 4.4HU, respectively.

When comparing absolute HU value differences for full‐fan acquisition techniques, FDK had similar performance as the iCBCT reconstruction (28.7 ± 32.6HU vs. 31.2 ± 39.5HU, respectively) between the two reconstruction techniques. However, when comparing the half‐fan techniques, iCBCT greatly improved HU value accuracy with FDK having an average HU value of 36.9 ± 42.5HU and iCBCT having an average HU value of 23.7 ± 33.1HU.

### 3.2 Dose calculation

For the head and neck phantom, across all objectives, the average percent difference between the CT‐sim plan and the CBCT was 0.2 ± 0.4% for FDK reconstructions and 0.2 ± 0.3% for the iCBCT reconstructions. The minimum and maximum percent differences were −1.0% to 1.1% and −0.5% to 1.0% for the FDK and iCBCT reconstructions, respectively. For the thorax phantom, deviations were larger between the two reconstruction techniques, where the difference between the CT‐sim plan and CBCT plan was −0.6 ± 3.1% for FDK and 0.2 ± 1.1% for the iCBCT reconstructions. The minimum and maximum percent differences were −11.7%–2.0% and −3.3%–2.3% for the FDK and iCBCT reconstructions, respectively. The lung SBRT treatment had the largest differences between the CT‐sim and CBCT plans where the difference was −11.7% for the PTV D98% for the FDK reconstruction and only −3.3% for the iCBCT reconstruction. On the thorax phantom, 83% and 98% of evaluated DVH metrics on FDK or iCBCT reconstructions, respectively, were within 3% of CT‐sim calculated across scan protocols. On the head phantom, all evaluated DVH metrics were within 3% of CT‐sim calculations. Full results are presented in Tables 3 and 4.

**TABLE 3:** Calculated dose–volume metrics across plan types, scan protocols, and reconstructions for the anthropomorphic thorax phantom.

Lung SBRT								
Treatment planning metrics	CT‐sim 120 kV	Thorax FDK 125 kV	Thorax iCBCT 125 kV	Pelvis FDK 125 kV	Pelvis iCBCT 125 kV	CT‐sim 140 kV	Pelvis Lg FDK 140 kV	Pelvis Lg iCBCT 140 kV
PTV D98% (Gy)	50.2	44.3	48.5	45.2	48.7	50.1	45.1	48.6
PTV D50% (Gy)	59.0	54.8	57.7	55.4	57.8	59.0	55.6	57.9
PTV D2% (Gy)	68.4	68.8	68.9	68.8	68.8	68.5	68.9	68.7
PTV D0.03cc (Gy)	68.8	69.4	69.2	69.3	69.1	68.8	69.3	69.1
Heart D0.03cc (Gy)	20.5	20.3	20.6	20.4	20.6	20.6	20.4	20.6
Cheswall D0.03cc (Gy)	21.2	21.4	21.3	21.4	21.3	21.1	21.4	21.3
Lung V20Gy (%Vol)	3.0	2.8	2.9	2.8	2.9	3.0	2.8	2.9
**Spine SBRT**								
PTV D98% (Gy)	26.4	26.5	26.9	26.7	26.8	26.7	26.9	26.7
PTV D50% (Gy)	29.3	29.3	29.7	29.6	29.6	29.5	29.7	29.5
PTV D2% (Gy)	34.3	34.3	34.8	34.7	34.7	34.6	34.8	34.7
PTV D0.03cc (Gy)	35.4	35.4	36.0	35.8	35.9	35.7	36.0	35.7
Heart D0.03cc (Gy)	15.4	15.3	15.4	15.5	15.3	15.4	15.4	15.4
Esophagus D0.03cc (Gy)	23.2	23.1	23.5	23.5	23.2	23.3	23.6	23.6
Cord D0.03cc (Gy)	21.1	21.2	21.4	21.4	21.4	21.3	21.4	21.4
**Rib SBRT**								
PTV D98% (Gy)	35.1	35.1	34.6	35.0	34.8	35.0	34.7	35.1
PTV D50% (Gy)	40.7	40.7	40.5	40.8	40.5	40.7	40.6	40.7
PTV D2% (Gy)	44.1	44.0	44.1	44.2	44.1	44.1	44.1	44.1
PTV D0.03cc (Gy)	44.3	44.2	44.4	44.4	44.3	44.3	44.3	44.4
Heart D0.03cc (Gy)	31.6	31.6	31.2	31.8	31.7	31.8	31.1	31.5
SkinRind D0.03cc (Gy)	38.1	38.1	38.6	38.1	38.4	38.3	38.5	38.2

Abbreviations: CBCT, cone beam computed tomography; CT, computed tomography; FDK, Feldkamp–Davis–Kress, IMRT, intensity‐modulated radiotherapy; SBRT, stereotactic body radiotherapy.

**TABLE 4:** Calculated dose–volume metrics across plan types, scan protocols, and reconstructions for the anthropomorphic head phantom.

Head and Neck IMRT					
Treatment planning metrics	CT‐sim 120 kV	Head FDK 100 kV	Head iCBCT 100 kV	Head* FDK 125 kV	Head* iCBCT 125 kV
PTV_High D98% (Gy)	69.5	69.7	69.8	69.6	69.8
PTV_High D50% (Gy)	72.2	72.4	72.4	72.3	72.3
PTV_High D2% (Gy)	74.6	74.8	74.7	74.6	74.7
PTV_High D0.03cc (Gy)	76.1	76.0	76.0	75.9	75.9
PTV_Mid D98% (Gy)	61.8	62.2	62.3	62.2	62.3
PTV_Mid D50% (Gy)	71.3	71.4	71.5	71.3	71.4
PTV_Mid D2% (Gy)	74.3	74.5	74.5	74.3	74.4
PTV_Low D98% (Gy)	54.3	54.5	54.5	54.5	54.5
PTV_Low D50% (Gy)	57.8	57.9	57.9	57.8	57.9
PTV_Low D2% (Gy)	60.4	60.5	60.5	60.5	60.5
Parotid_L Dmean (Gy)	25.8	26.0	26.0	26.0	26.0
Parotid_R Dmean (Gy)	29.8	29.6	29.8	29.8	29.9
Cord D0.03cc (Gy)	41.0	41.3	41.2	41.1	41.2
**Hippocampal avoidance IMRT**					
PTV D98% (Gy)	26.2	26.2	26.2	26.2	26.2
PTV D50% (Gy)	32.6	32.7	32.7	32.7	32.7
PTV D2% (Gy)	35.8	35.8	35.8	35.8	35.8
PTV D0.03cc (Gy)	37.9	38.1	38.0	38.0	38.0
Hippocampus D0.03cc (Gy)	16.2	16.1	16.1	16.1	16.2
**Brain SRS**					
PTV D98% (Gy)	27.0	27.1	27.1	26.9	27.1
PTV D50% (Gy)	32.5	32.7	32.6	32.6	32.5
PTV D2% (Gy)	35.4	35.7	35.5	35.5	35.5
PTV D0.03cc (Gy)	35.4	35.6	35.5	35.5	35.5
Brain‐GTV D15cc (Gy)	5.8	5.9	5.9	5.9	5.9

Abbreviations: CBCT, cone beam computed tomography; CT, computed tomography; FDK, Feldkamp–Davis–Kress, IMRT, intensity‐modulated radiotherapy; SRS, stereotactic radiosurgery.

For the 1%/2 mm gamma analysis, the iCBCT reconstruction comparisons were > 97.7% for both the head and custom head protocols. The FDK reconstructions for this treatment site were similar, with 98.5% and 98.6% for the head and custom head protocols, respectively. For the thorax phantom, 7 out of 9 of the FDK reconstructions did not have a gamma analysis of 100.0%. The thorax protocol with FDK reconstruction performed the worst at 98.6%, followed by pelvis FDK (99.6%) and pelvis large (99.7%).

**FIGURE 2:**
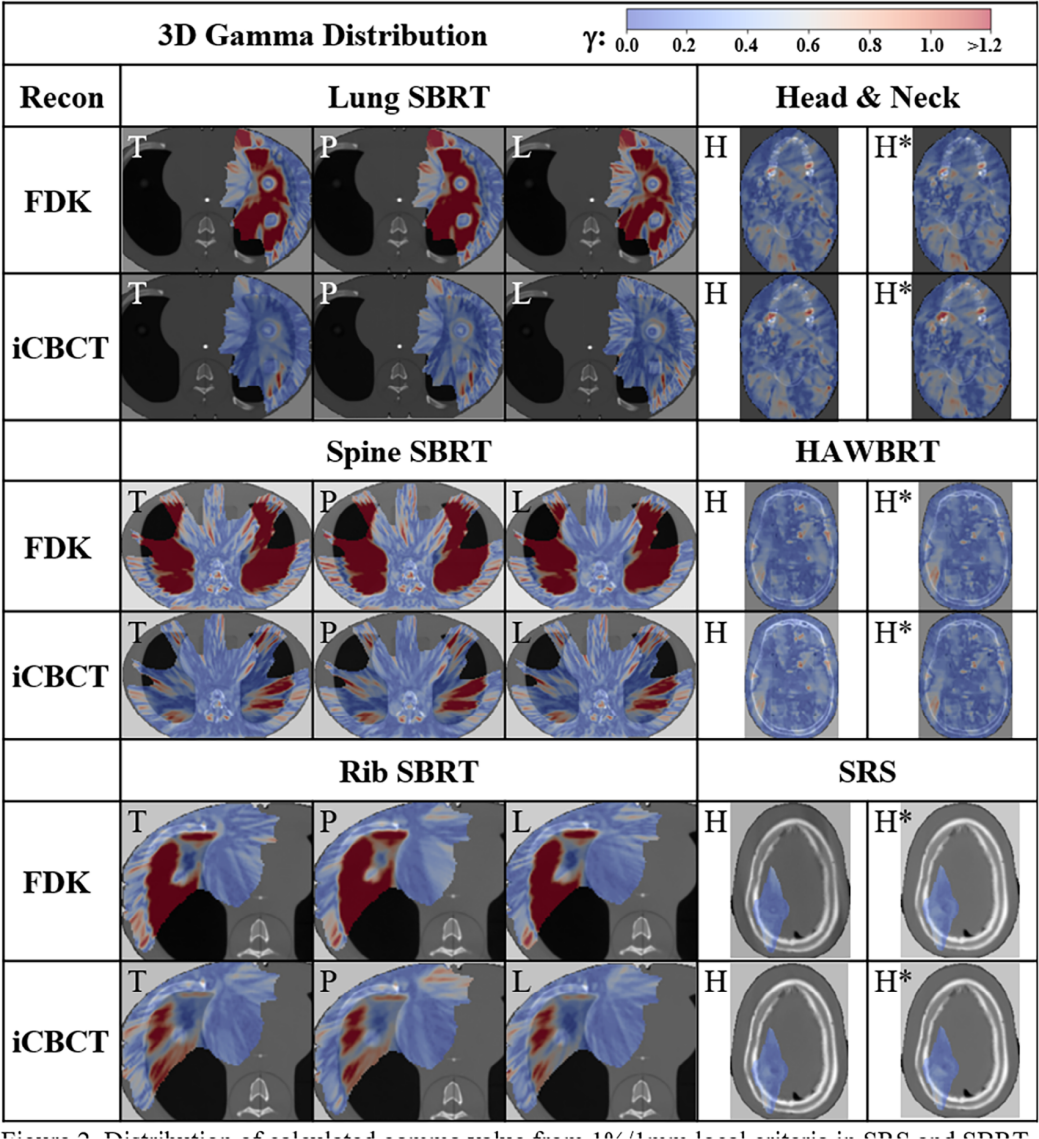
Distribution of calculated gamma value from 1%/1 mm local criteria in SRS and SBRT or 1%/2 mm for IMRT and 10% dose thresholding in the axial slice containing maximum dose across body sites, protocol (T = Thorax, P = Pelvis, L = Pelvis Large, H = Head, and H^*^= Head with custom parameters) and reconstruction methods. IMRT, intensity‐modulated radiotherapy; SBRT, stereotactic body radiotherapy; SRS, stereotactic radiosurgery.

For the 1%/1 mm gamma analysis, the difference in results was more substantial for the thorax phantom compared to the head phantom. The SRS plan on the head phantom had a gamma pass rate of 100% for all images, whereas the poorest performing gamma analysis for the thorax phantom was 69.7% for the spine SBRT plan using the pelvis protocol and FDK reconstruction. In comparison, that same plan reconstructed with the iCBCT method was 94.2%. In total, the average gamma analysis for the head and neck phantom was 99.1 ± 0.9% and 99.1 ± 0.9% for the FDK and iCBCT protocols, respectively. For the thorax phantom, the average gamma analysis was 78.0 ± 6.1% and 96.6 ± 2.3% for the FDK and iCBCT protocols, respectively (Table 5). Gamma analysis 2D plots for the analysis are illustrated in Figure 2.

**TABLE 5:** Calculated local gamma pass percentages with 10% dose thresholding by treatment site and image acquisition. SBRT and SRS plans used 1%/1 mm criteria, while IMRT plans used 1%/2 mm criteria.

Plan	Thorax FDK	Thorax iCBCT	Pelvis FDK	Pelvis iCBCT	Pelvis Large FDK	Pelvis Large iCBCT
**Spine SBRT**	73.0	94.0	69.7	94.2	75.8	93.4
**Rib SBRT**	83.9	94.7	84.4	97.3	88.2	98.1
**Lung SBRT**	77.3	99.6	70.6	99.4	79.0	98.4

**Plan**	**Head FDK**	**Head iCBCT**	Head* FDK	Head* iCBCT
**HAWBRT**	99.4	99.5	99.5	99.5
**H&N**	97.9	97.8	97.8	97.8
**SRS**	100	100	100	100

Abbreviations: CBCT, cone beam computed tomography; CT, computed tomography; FDK, Feldkamp–Davis–Kress, IMRT, intensity‐modulated radiotherapy; SBRT, stereotactic body radiotherapy, SRS, stereotactic radiosurgery.

## 4 DISCUSSION

### 4.1 HU characterization

This work successfully evaluated the impact of a C‐arm linac equipped with an improved CBCT imaging system in combination with advanced image registration algorithms on HU accuracy and dose calculation. Results demonstrated that the HyperSight CBCT enables high HU accuracy and precision in phantoms leading to improvements in dose calculation accuracy. The combination of an advanced imager in conjunction with advanced image reconstruction algorithms was needed for these HU accuracy improvements. These findings suggest that more efficient workflows can now be enabled to handle online and offline changes needed during radiotherapy when using a C‐arm linac.

CBCTs have higher HU uncertainty compared to FBCT imaging due to acquisition geometry that results in more scatter contribution.^9^ This results in less accurate HU values and contributes to a higher uncertainty of HU values for a particular density, which may lead to less accurate dose calculations.^23^ This study demonstrates these physical differences, since the standard deviation was higher in the CBCT HU values when compared to CT‐sim. Overall, advanced reconstruction and half‐fan techniques improved the accuracy and precision of HU values over FDK and full‐fan techniques. In this study, FDK reconstructions resulted in marginally better accuracy for some higher‐density materials for the single customized scan protocol, Head*. This could be due to the scatter correction kernel in iCBCT‐MAR not accounting for these high densities—a topic for future investigations.

In a prior version of this CBCT imaging system, iCBCT reconstruction also had a substantial impact on HU values but was still variable in its accuracy, demonstrating the necessity of the advanced imaging panel and reconstruction techniques for proper HU accuracy.^24^ However, as the density increased, the HU values began to deviate from the CT‐sim. This was also demonstrated using this same advanced imaging system on an O‐ring linac.^14^ In that investigation, the authors demonstrated substantially worse deviations in HU value using a C‐arm linac in comparison to an O‐ring unit, thus indicating that the advanced imager with advanced reconstructions limits these deviations. Despite the large discrepancies in CT number at higher densities, the vast majority of a patient's anatomy is near‐water density, thus isolating areas of inaccurate dose calculations to high‐density bone or metal implants. This is a small subset of the patient population treated within radiotherapy and may be clinically inconsequential.

The report of TG‐66 requires annual constancy checks of the CT Number accuracy for density correction in treatment planning to within 5 HU using the same phantom configuration as baseline, with the same tolerance for daily HU check for true water.^1^ The high standard deviations calculated in materials using the advanced CBCT suggest variance from the recommendations of the TG‐66. However, these investigations were carried out under several scattering conditions, which contributes to the variation in HU value. Although the constancy of this imaging system over time is not investigated in this work, future studies should indicate quality measures for ensuring stability of HU values. Additionally, despite CT number accuracy deviations from CT‐sim, these results may still be clinically acceptable since HU values are not expected to be universal across imaging systems due to varied x‐ray energy spectrums.

### 4.2 Dose calculation

When evaluating the plan calculation results, the HyperSight imaging system with advanced image reconstruction algorithms presents limited differences in clinically important dosimetric goals for the half‐fan techniques. The use of an advanced reconstruction technique appeared to have minimal impact on the head and neck phantom, which had less heterogeneities and was closer to near‐water densities. Additionally, calculating in more homogenous near‐water density locations is robust to dose calculation errors even without advanced imaging or reconstruction techniques.^14^, ^25^ In the thorax phantom, the advanced reconstruction algorithm was successful in creating clinically acceptable plans, considering that all but a single (0.9%) dose objective evaluated would have not passed MPPG5b guidance on evaluating dose calculation accuracy.^20^ The only objective to fail the 3% criteria was the PTV D98% of the lung SBRT plan in the buildup region, where dose calculations are generally less accurate.^26^ Despite higher densities deviating from the CT‐sim HU‐values, the impact on the dose objectives for the spine and rib SBRT was minimal. Others have demonstrated with an O‐ring linac similar findings where minimal differences were seen in dose calculation comparisons in the presence of higher density values.^23^

To date, there have not been gamma evaluations comparing the dose calculation on a C‐arm linac equipped with an advanced imaging system. Since geometric properties between this system and an O‐ring are different, investigation of C‐arm on‐board imaging systems for dose calculation is essential. The gamma analyses performed in this work demonstrated that the advanced imager with advanced image reconstruction algorithms performs well and is similar to results demonstrated by groups with O‐ring linacs.^23^ The restrictive 1%/1mm gamma results were comparable to the 2%/2 mm gamma evaluation results in a previous version of the CBCT imaging system on an O‐ring linac (98.0%).^27^ In the study by Hu et al. plans were calculated with Acuros XB (AXB) dose algorithm for comparison to CT‐sim while the current study calculated with CCC. Therefore, the difference in dose calculation algorithms used may introduce error for comparison between these two studies. With the FDK reconstruction, suboptimal gamma results were calculated specifically for heterogeneous regions and low densities, which is where larger deviations from CT‐sim HU values in the physical density curve occured. As observed with the dosimetric objectives in the head and neck phantom acquired with the full‐fan technique, the reconstruction algorithm appeared to have minimal impact on the gamma analysis results. Therefore, the iCBCT algorithms appear to have a more substantial and positive impact on the half‐fan techniques compared to the full‐fan techniques, albeit the full‐fan techniques have acceptable results with either reconstruction algorithm.

When comparing with previous versions of the vendor's CBCT imaging systems, we see challenges with an HU value accuracy of both lower and higher densities. Rong et al. reported the difference in CBCT HU values at low density values of up to 250 HU when compared to CT HU values.^28^ When iCBCT was introduced on the prior version of the imaging system evaluated in this study, Washio et al demonstrated that there were higher deviations on average of 100 HU for high–density values between protocols compared to our study, which was 50–75 HU.^29^ Therefore, we conclude the HU value accuracy is improved in the imaging system evaluated in this study compared to previous versions.

Current radiation oncology workflows requiring conventional CT‐sim increase the time to treatment start or replan start. CBCTs that approach CT‐sim quality may result in a net benefit to patients due to increased efficiency in workflow.^30^, ^31^ This could be most immediately realized in emergent palliative treatments for spinal cord compressions, removing the pain induced during the transfer onto and off the CT‐sim imaging couch.^32^ Additionally, offline replans for head and neck treatments may also benefit from these advanced imagers to avoid the need for an additional CT‐sim to accelerate the turnaround time and avoid complicated logistics for re‐simulation.^33^

Limitations of this work include the lack of dose calculation impact around metal implants or including the impact of motion artifacts, which are present in vivo and have an impact on dose calculation accuracy.^13^ There may be local dose calculation effects due to patient‐specific scattering conditions or heterogeneities within the treatment volume that may exacerbate dose calculation errors due to these increased complexities. Therefore, commissioning CBCT dose calculation by disease site may be necessary to evaluate where uncertainties exist in vivo and treatment plan accordingly to minimize or respect the potential discrepancies in dose calculation. Further studies are needed to assess these limitations.

Analyses were also performed to examine voxel‐wise HU difference impact on dose calculation, included in the Supporting Information [Link], [Link], [Link], [Link], [Link], [Link]. The results suggest that HU differences in a single voxel yield little to no impact on the dose calculation error in that same voxel. We hypothesize that consistent and large‐volume HU differences will change dose more substantially as the radiation beam travels and interacts across many voxels.

## 5 CONCLUSION

This work demonstrated that an advanced imager with advanced image reconstruction algorithms enables accurate direct‐to‐CBCT dose calculation in phantom. DVH metrics calculated in heterogeneous, anthropomorphic phantoms reported using the novel reconstruction method across most acquisition techniques is within the 3% tolerance for heterogeneous TPS validation distal to lung tissue established by MPPG5b and 5% for secondary dose calculations in heterogeneous mediums at high dose/low gradient established by TG‐219 when comparing to calculations on conventional CT, with the exception of D98 calculations on the low‐exposure thorax protocol.^20^ 3D gamma was used to assess dose distributions of the novel imaging techniques and reconstructions against CT‐sim and found minimum 93.4% voxels passed despite sensitive gamma criteria. Furthermore, the consistent and robust CT number analysis across many arrangements suggest these images may be useful for body sites or situations outside the scope of the current work. This will have positive implications for the field of adaptive radiotherapy in both online and offline settings as C‐arm linacs remain the predominant machine used within radiotherapy.

## AUTHOR CONTRIBUTIONS

Kenneth Gregg designed experiments, collected data, performed analysis, created figures, and intellectually contributed to the writing of the manuscript. Theodore Arsenault designed experiments, aided in figure creation, data analysis, and intellectually contributed to the writing of this manuscript. Atefah Rezaei, Rojano Kashani, and Lauren Henke were involved in the conception and design of experiments and intellectually contributed to the writing of this manuscript. Alex Price designed experiments, collected data, performed analysis, created figures, and intellectually contributed to the writing of this manuscript.

## CONFLICT OF INTEREST STATEMENT

Kenneth Gregg, Theodore Arsenault, Atefeh Rezaei, and Rojano Kashani report no conflicts of interest related to this project. Alex Price has received funding from Radformation for giving research presentations. Lauren Henke has received funding from Varian Medical Systems for consulting and giving research presentations and is on the Elekta medical advisory board. University Hospitals was funded with an industry grant by Varian Medical Systems.

## Supporting information

Supporting Information

Supporting Information

Supporting Information

Supporting Information

Supporting Information

Supporting Information

Supporting Information
