# Supplementary figures and images for: Hounsfield Unit characterization and dose calculation on a C‐arm linac with novel on‐board cone‐beam computed tomography feature and advanced reconstruction algorithms
Source: J Appl Clin Med Phys. 2025 Jul 25;26(8):e70145. doi: 10.1002/acm2.70145 (PMC12301083; doi:10.1002/acm2.70145)

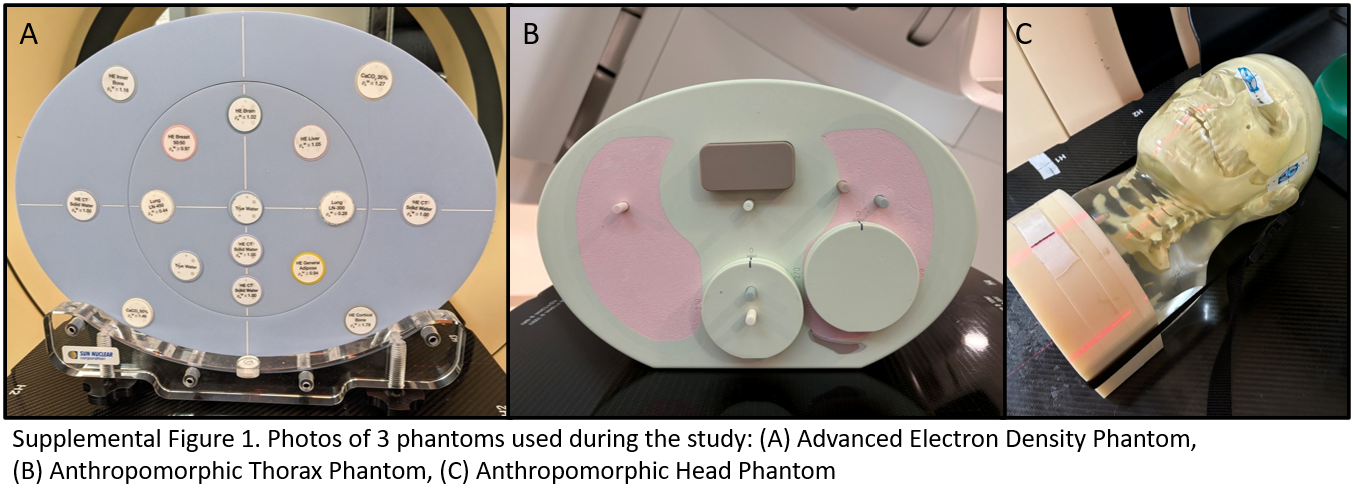

Supplement: Supplementary file 1 — Supporting Information [file ACM2-26-e70145-s001.png]

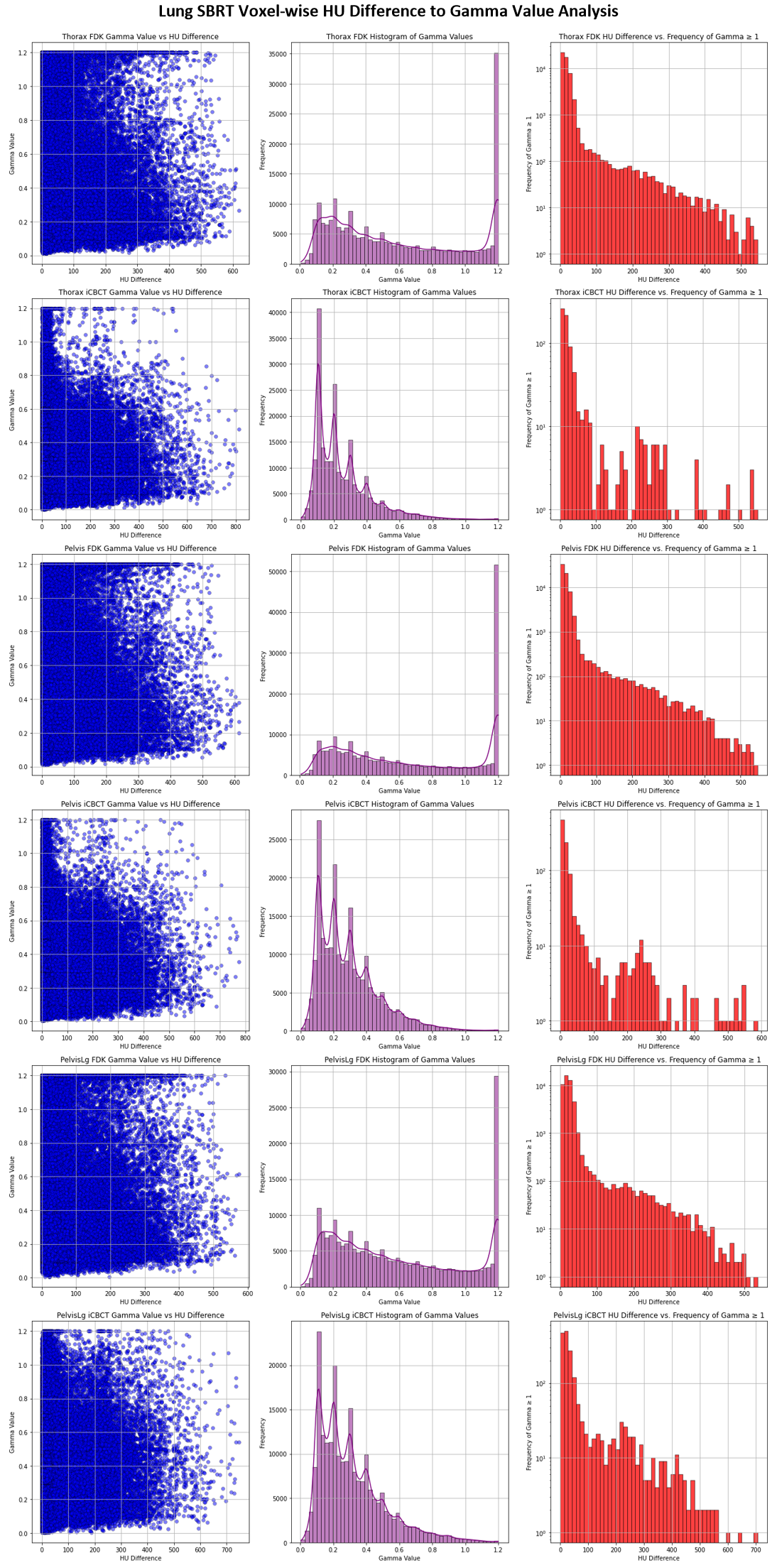

Supplement: Supplementary file 2 — Supporting Information [file ACM2-26-e70145-s005.png]

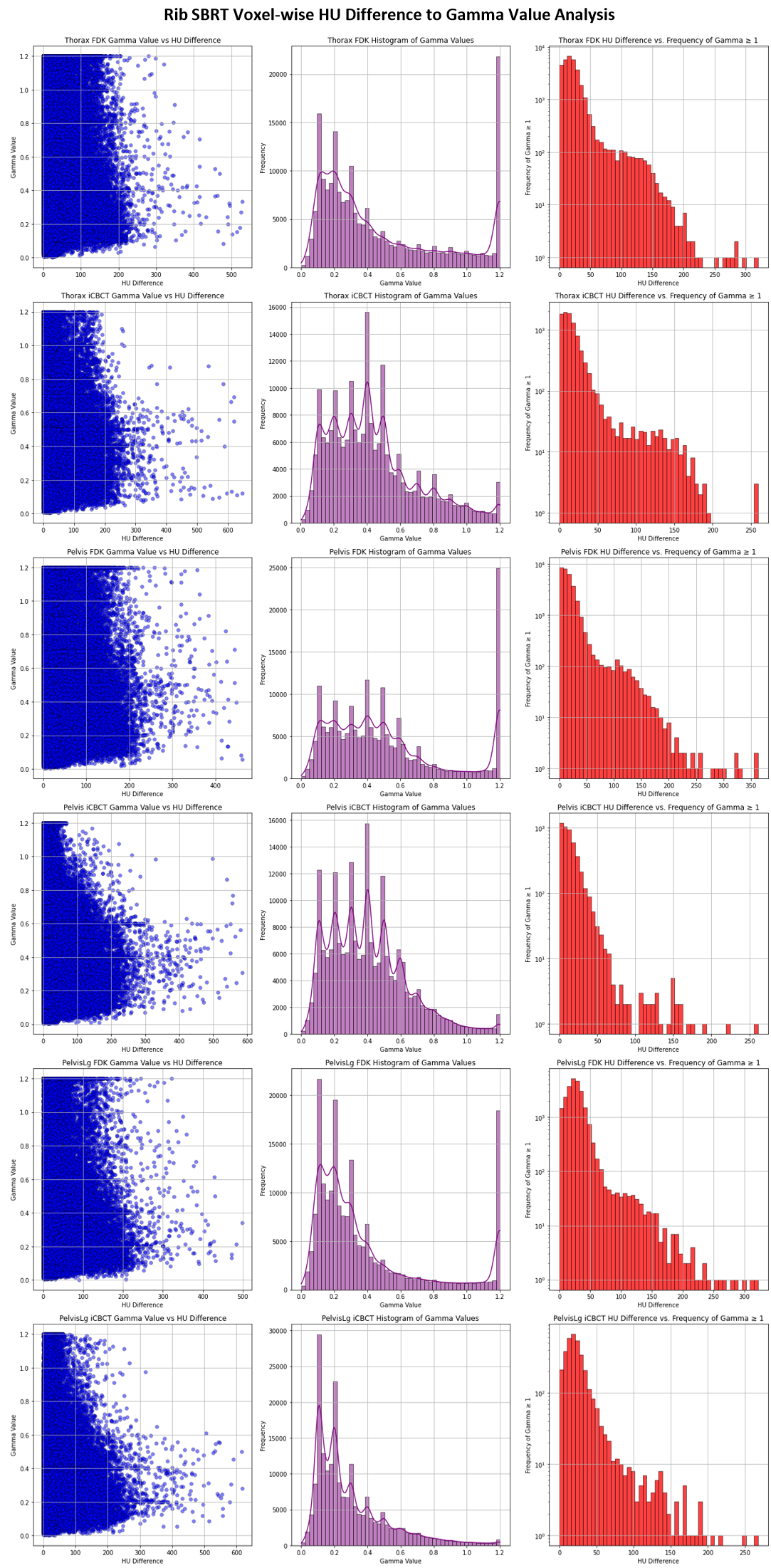

Supplement: Supplementary file 3 — Supporting Information [file ACM2-26-e70145-s004.png]

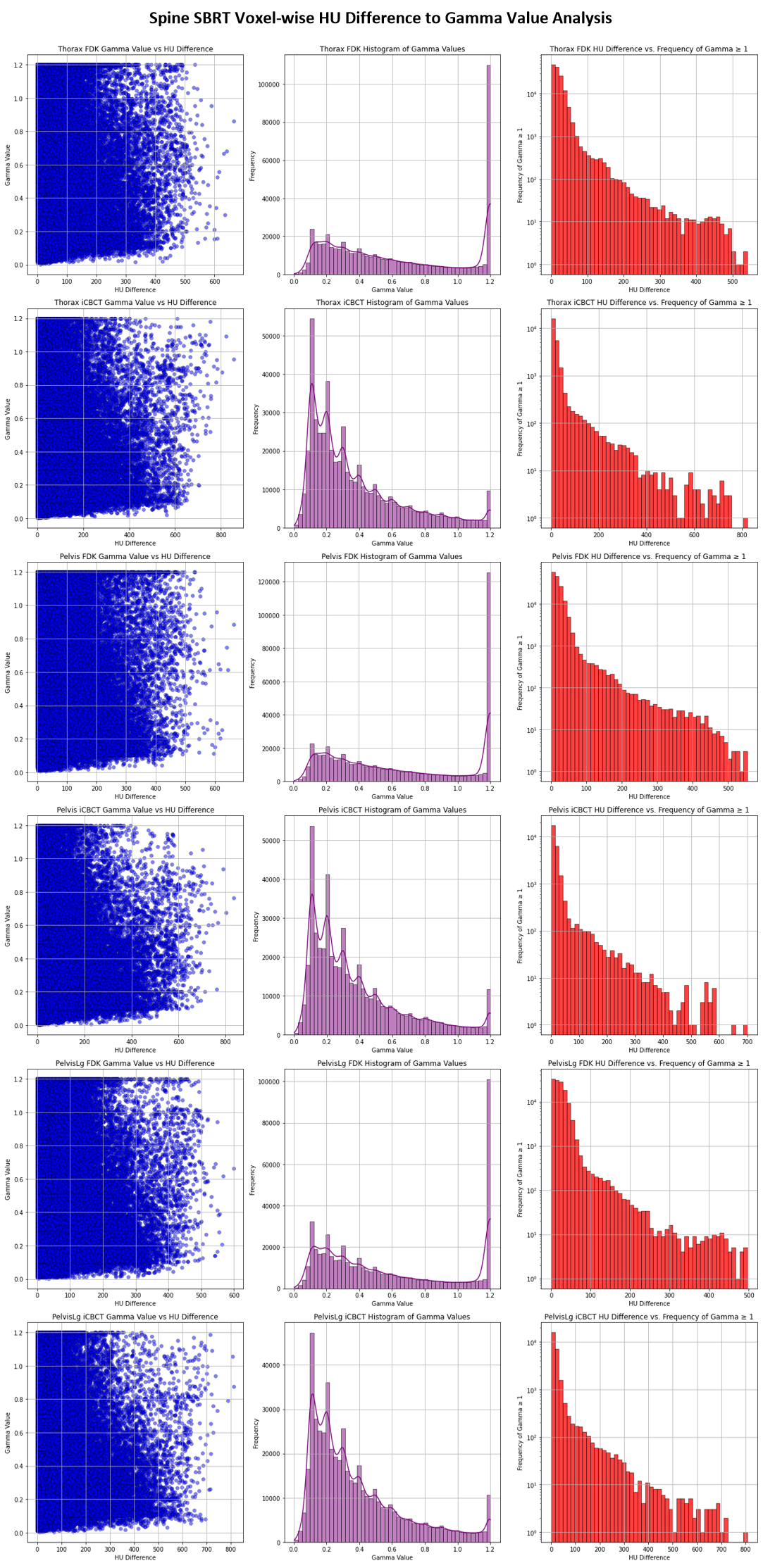

Supplement: Supplementary file 4 — Supporting Information [file ACM2-26-e70145-s002.png]

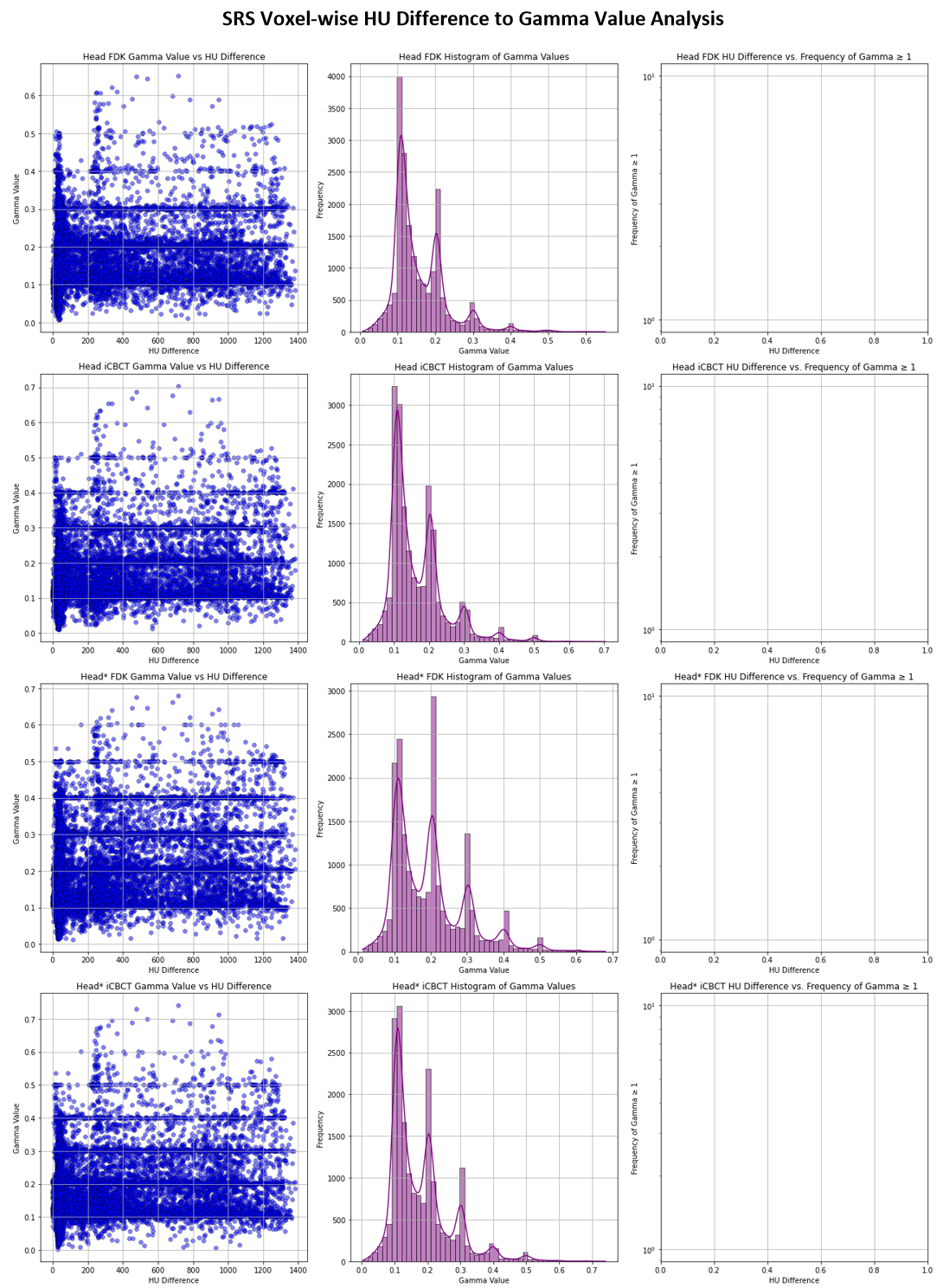

Supplement: Supplementary file 5 — Supporting Information [file ACM2-26-e70145-s007.png]

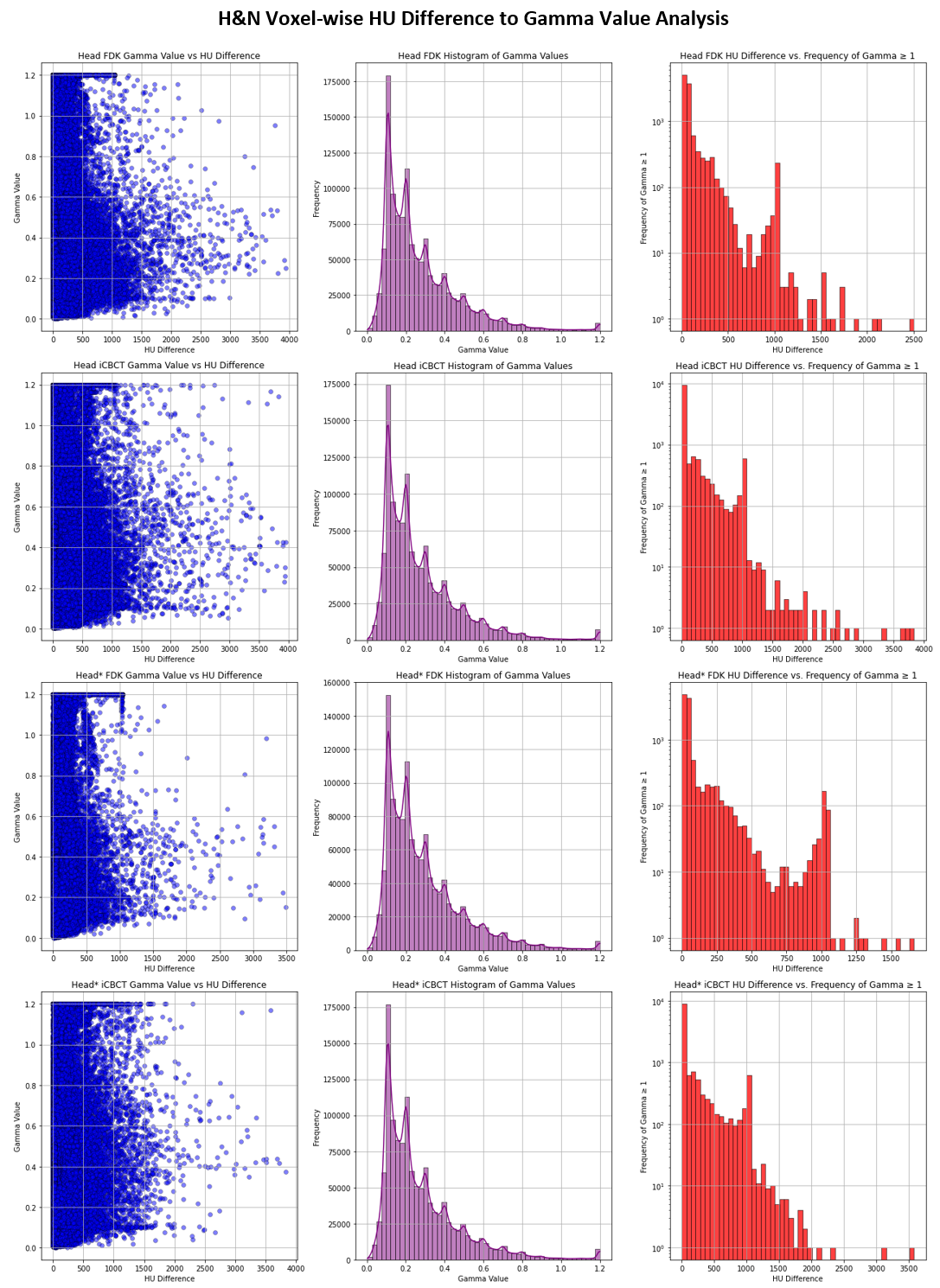

Supplement: Supplementary file 6 — Supporting Information [file ACM2-26-e70145-s006.png]

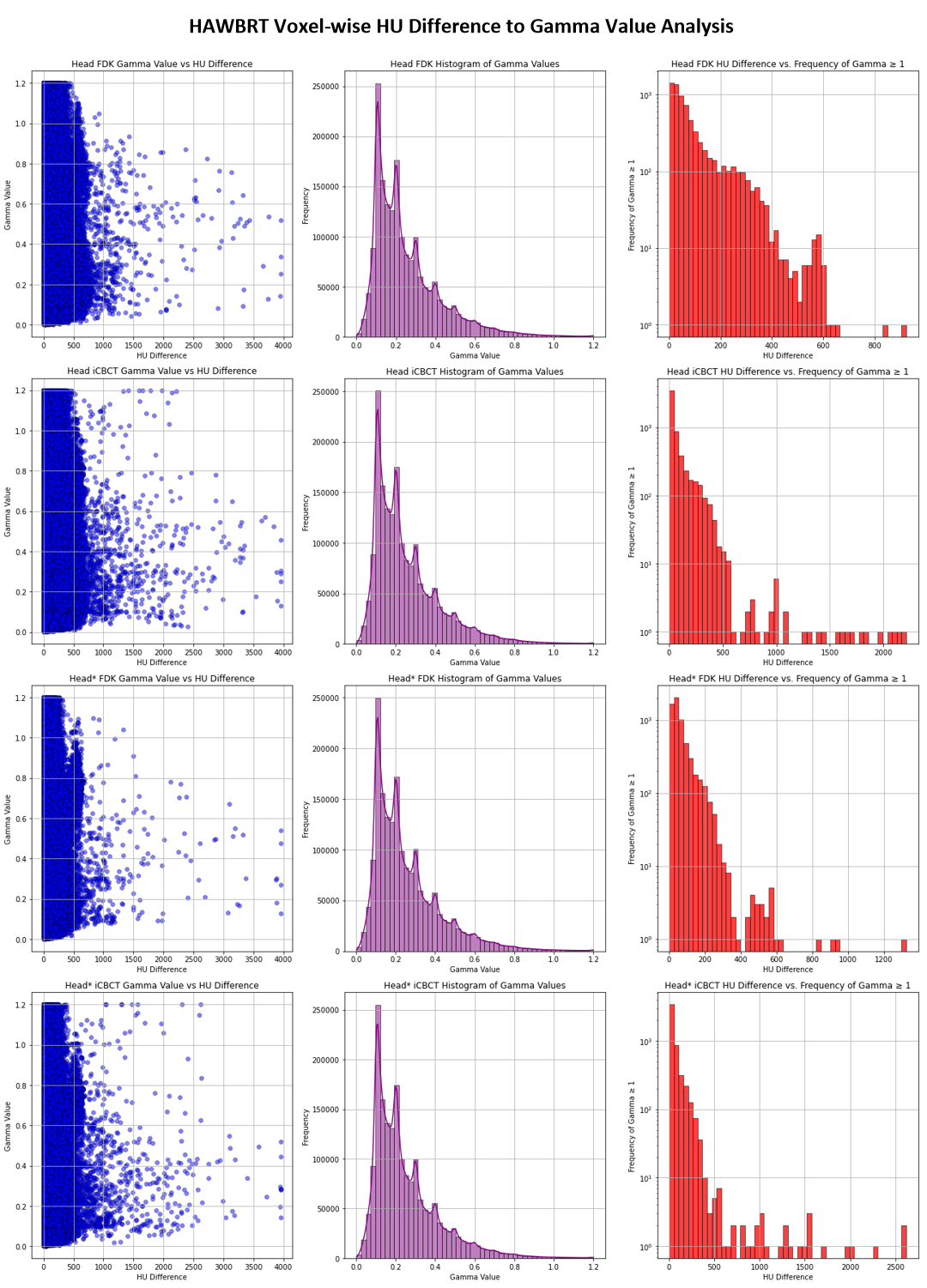

Supplement: Supplementary file 7 — Supporting Information [file ACM2-26-e70145-s003.png]
